# Bilateral Hearing Loss and Tinnitus as Primary Manifestations of Pontine Hemorrhage in a Young Man With Refractory Hypertension

**DOI:** 10.7759/cureus.91733

**Published:** 2025-09-06

**Authors:** Zhuo Luan, Aleksandr Drozdov, Jithendhar Kandimalla

**Affiliations:** 1 Neurology, Texas Tech University Health Sciences Center El Paso, EL Paso, USA; 2 Radiology, Texas Tech University Health Sciences Center El Paso, El Paso, USA

**Keywords:** auditory pathway, bilateral hearing loss, hypertension, pontine hemorrhage, young adults

## Abstract

Pontine hemorrhages are typically associated with profound neurological deficits such as coma and quadriparesis, but selective involvement of the dorsal tegmentum can produce more subtle or atypical symptoms. We report the case of a 32-year-old man with longstanding, treatment-noncompliant hypertension who presented with acute-onset bilateral hearing loss and tinnitus. Head CT revealed an acute hemorrhage localized to the pontine tegmentum, yet the patient remained fully awake and alert. Renovascular causes of hypertension were excluded, but the workup revealed evidence of chronic hypertensive end-organ damage, including hypertensive retinopathy, renal parenchymal disease, and severe concentric left ventricular hypertrophy. This case underscores the importance of considering posterior circulation events in hypertensive patients, particularly young adults, presenting with new bilateral auditory symptoms, as early neuroimaging and recognition of atypical presentations are critical for accurate diagnosis and timely intervention.

## Introduction

Pontine hemorrhages, while accounting for only a small fraction of intracerebral hemorrhages (approximately 7.5% of all hemorrhagic strokes, with an incidence of about 3 per 100,000 people annually), often carry a poor prognosis due to involvement of critical brainstem structures [1]. Although pontine hemorrhages typically present with coma, gaze palsy, or quadriparesis, smaller or tegmental lesions may manifest with atypical findings depending on the neuroanatomy involved [2,3]. The dorsal tegmentum of the pons contains essential auditory pathways, including the cochlear nuclei and decussating auditory fibers [4], and hemorrhage involving this area may result in hearing impairment. Bilateral hearing loss and tinnitus as the primary manifestations of pontine hemorrhage are exceedingly rare, with only a few reported cases [5,6], and may be easily misattributed to peripheral causes, especially in young patients. In such cases, delayed diagnosis can lead to missed opportunities for early intervention and may be fatal if the hemorrhage expands. Here, we present a unique case of pontine tegmental hemorrhage in a young man with longstanding refractory hypertension, presenting primarily with bilateral hearing loss and tinnitus.

## Case presentation

A 32-year-old man with refractory hypertension presented to the emergency department with sudden-onset bilateral hearing loss and constant, nonpulsatile tinnitus, accompanied by nonspecific numbness and tingling throughout his body. He described a sensation of “muffled ears” and difficulty hearing. He denied headache, vertigo, nausea, weakness, facial droop, or new visual changes. He had a prior diagnosis of hypertensive retinopathy following left eye vision loss. He denied current or recent drug use but reported a remote history of cocaine use. On arrival, vital signs showed a blood pressure of 262/182 mmHg. Neurologically, he was fully alert and oriented. Cranial nerve examination was normal except for impaired hearing and remote left eye vision loss. No facial asymmetries or motor deficits were noted. The examiner was required to speak in a very loud voice to effectively communicate with the patient.

**Figure 1:**
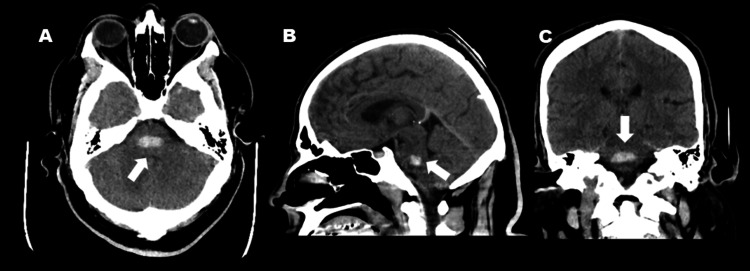
Acute hemorrhage in the pons. The axial (A) and sagittal (B) and coronal (C) of the initial brain computed tomography showed an acute phase hemorrhage (2.1 cm x 9 mm x 1.1 cm) of the pontine tegmentum (arrows).

**Figure 2:**
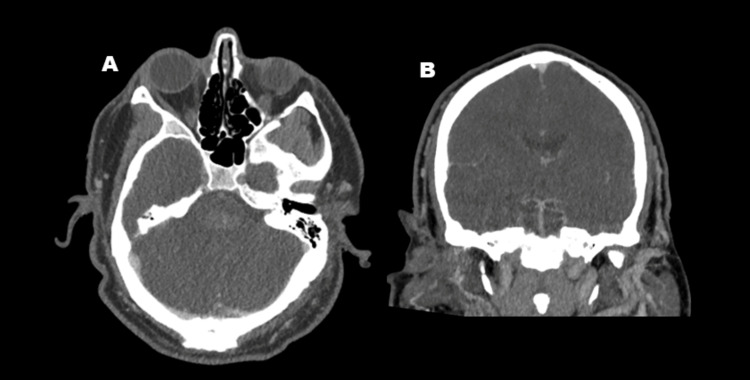
No vascular abnormalities in posterior circulation. The axial (A) and coronal (B) of CT angiogram showed no vascular malformations or stenosis.

**Figure 3:**
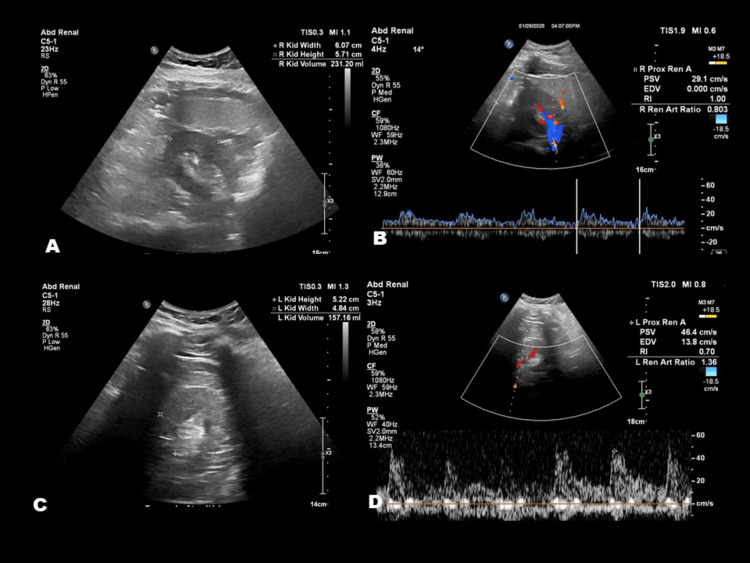
Bilateral renal ultrasound. Bilateral renal ultrasound demonstrates increased echogenicity of the renal cortices, consistent with medical renal disease (A: right; C: left). No evidence of renal artery stenosis is noted bilaterally (B: right; D: left).

Non-contrast head CT at admission revealed an acute, oval-shaped hemorrhage centered in the dorsal pons within the tegmentum, measuring 2.1 × 0.9 × 1.1 cm (Figure 1). CT angiography of the head and neck showed no evidence of vascular malformations or stenosis (Figure 2). Laboratory studies demonstrated a negative urine drug screen, elevated plasma renin activity with normal aldosterone levels, and hyperkalemia (Table 1). Serum creatinine was mildly elevated, and urinalysis revealed significant proteinuria. Renal ultrasound showed bilaterally increased echogenicity, suggestive of chronic parenchymal disease, without evidence of renal artery stenosis (Figure 3). An echocardiogram revealed severe concentric left ventricular hypertrophy with preserved ejection fraction (not shown). Additional workup for secondary hypertension, including evaluation for pheochromocytoma and hyperthyroidism, was negative, as plasma metanephrines, normetanephrines, TSH (thyroid-stimulating hormone), T3, and T4 levels were within normal limits (Table 1). Overall, the evaluation excluded renovascular and endocrine causes of hypertension but revealed clear evidence of chronic hypertensive end-organ damage, including hypertensive retinopathy, renal parenchymal disease, and severe concentric left ventricular hypertrophy.

**Table 1:** Laboratory results at initial presentation. Laboratory studies demonstrated hyperkalemia and elevated plasma renin activity with normal aldosterone levels. And evaluation for pheochromocytoma and hyperthyroidism, was negative, as plasma metanephrines, normetanephrines, TSH, T3, and T4 levels were within normal limits. CBC: complete blood count; WBC: white blood cells; RBC: red blood cells; HGB: hemoglobin; PLT: platelets; CMP: comprehensive metabolic panel; BUN: blood urea nitrogen; AST: aspartate aminotransferase; ALT: alanine aminotransferase; ALK phos: alkaline phosphatase; T4: thyroxine; T3: triiodothyronine; TSH: thyroid-stimulating hormone; MIU/L: milli–international units per liter; MN: metanephrine; NMN: normetanephrine.

	Blood/Serum	Admission	Reference
CBC	WBC	9.82	4.5-11 x103/uL
	RBC	5.48	3.5-5.5 x106/uL
	HGB	15.8	12-15 g/DL
	PLT	333	150-450 x103/uL
CMP	Sodium	139	135-145 mmol/L
	Potassium	3.0	3.5-5.1 mmol/L
	BUN	11	7-17 mg/dL
	Creatinine	1.1	0.52-1.04 mg/dL
	Total bilirubin	0.9	0.2-1.3 mg/dL
	AST	35	14-36 IU/L
	ALT	60	0-35 IU/L
	ALK phos	70	38-126 IU/L
Other labs	Aldosterone	3	3-28 ng/dL
	Metanephrine, free	<25	<57 pg/mL
	Normetanephrine, free	113	<148 pg/mL
	Total, free (MN + NMN)	113	<205 pg/mL
	T4, free	1.36	0.78-2.19 ng/dL
	T3, free	4.17	2.77-5.27 ng/dL
	TSH	2.35	0.47-4.68 MIU/L
	Plasma renin activity	7.31	0.25-5.82 ng/mL/h

At admission, he was admitted to the Neuro-ICU for blood pressure control with intravenous clevidipine, targeting a systolic blood pressure of less than 150 mmHg. Two days later, he was gradually transitioned to oral therapy and ultimately required four oral antihypertensive agents: amlodipine 10 mg daily, carvedilol 12.5 mg twice daily, clonidine 0.2 mg three times daily, and hydrochlorothiazide 50 mg daily. He was scheduled for follow-up with his primary care physician in four weeks for optimization of antihypertensive therapy. He remained neurologically stable throughout his hospitalization. On hospital day seven, at the time of discharge, he continued to experience hearing difficulty, although the tinnitus and paresthesia had resolved. He was discharged with outpatient follow-up.

## Discussion

This case illustrates an uncommon presentation of pontine hemorrhage in which the dominant clinical features were bilateral hearing loss and tinnitus, without classic signs of brainstem stroke such as gaze palsy, weakness, or altered mental status [2]. The localization of the hemorrhage to the pontine tegmentum explains the auditory symptoms, as this region contains critical components of the central auditory pathway.

Auditory signals are transmitted via the cochlear nerves, which relay to the cochlear nuclei located at the pontomedullary junction. Secondary auditory fibers decussate through the trapezoid body and ascend bilaterally via the lateral lemniscus to the inferior colliculi [7]. Lesions involving the tegmental pons, particularly if midline or bilateral, can disrupt this pathway and result in bilateral hearing impairment [8,9]. While bilateral cortical lesions can also affect auditory perception, brainstem lesions are more likely to cause tinnitus and sensorineural hearing loss [10]. Such findings provide valuable localizing information, highlighting the clinical significance of auditory deficits in identifying pontine or brainstem pathology. In the context of pontine hemorrhage, acute-onset bilateral hearing loss and tinnitus may serve as a rare but specific clinical indicator of tegmental involvement, allowing clinicians to correlate the patient’s auditory symptoms with the site of vascular injury and guide management strategies.

Pontine hemorrhages are most commonly caused by hypertensive microvascular rupture, typically involving small perforating arteries from the basilar artery. In this patient, chronic malignant hypertension was the likely etiology. Elevated renin and renal parenchymal changes suggest secondary hyperreninemia from hypertensive nephrosclerosis. The absence of renovascular or endocrine causes supports a diagnosis of primary hypertension with significant end-organ damage. Although uncommon, cocaine use is a known risk factor for both ischemic and hemorrhagic stroke, particularly in young individuals [11]. Even remote exposure can contribute to vascular remodeling and arteriopathy [12], which may increase hemorrhagic risk in the setting of uncontrolled hypertension [13].

This case underscores the importance of recognizing atypical stroke presentations. In posterior circulation events, symptoms such as hearing loss, vertigo, or sensory disturbances may precede more obvious neurological deficits. Stroke and emergency clinicians must maintain a high index of suspicion and pursue prompt neuroimaging, especially in hypertensive patients or those with other cerebrovascular risk factors.

## Conclusions

Bilateral hearing loss and tinnitus are rare but significant warning signs of brainstem pathology. In hypertensive patients, especially young adults with signs of end-organ damage, these symptoms should prompt immediate neuroimaging to assess for posterior circulation hemorrhage. This case highlights the need for early recognition of atypical stroke presentations and the importance of aggressive hypertension management to prevent devastating outcomes.
